# Synthesis of Nb-Doped TiO_2_ Nanoparticles for Photocatalytic Degradation of Ciprofloxacin: A Combined Experimental and DFT Approach

**DOI:** 10.3390/nano15171307

**Published:** 2025-08-25

**Authors:** Bouthaina Shili, Othmen Khaldi, Cristian Mendes-Felipe, Maibelin Rosales, Dinis C. Alves, Pedro M. Martins, Rached Ben Younes, Senentxu Lanceros-Mendez

**Affiliations:** 1 Laboratory of Technology Energy and Innovative Materials, Department of Physics, Faculty of Sciences of Gafsa, University of Gafsa, Gafsa 2112, Tunisia; bouthaina.shili@ugaf.tn (B.S.); rached.benyounes@fsgf.rnu.tn (R.B.Y.); 2 Laboratory of Materials Organization and Properties LMOP (LR99ES17), Faculty of Sciences of Tunis El Manar, University of Tunis El Manar, Tunis 2092, Tunisia; othmen.khaldi@fsgf.ugaf.tn; 3 BCMaterials—Basque Center for Materials, Applications and Nanostructures, UPC/EHU Science Park, 48940 Leioa, Spain; 4 Centre of Physics of Minho and Porto Universities (CF-UM-UP), Laboratory of Physics for Materials and Emergent Technologies, LapMET University of Minho, 4710-057 Braga, Portugal; id11657@alunos.uminho.pt; 5 Centre of Molecular and Environmental Biology (CBMA), University of Minho, 4710-057 Braga, Portugal; pamartins@bio.uminho.pt; 6 Institute for Research and Innovation on Bio-Sustainability (IB-S), University of Minho, 4710-057 Braga, Portugal; 7 IKERBASQUE, Basque Foundation for Science, 48009 Bilbao, Spain

**Keywords:** photocatalyst, Nb-doped TiO_2_, Density Functional Theory (DFT), nanomaterials, ciprofloxacin degradation, electronic structure modification

## Abstract

The persistence of pharmaceutical pollutants such as ciprofloxacin (CIP) in aquatic environments represents a critical environmental threat due to their potential to induce antimicrobial resistance. Photocatalysis using TiO_2_-based materials offers a promising solution for their mineralization; however, the limited visible-light response of TiO_2_ and charge carrier recombination restricts its overall efficiency. In this study, Nb-doped TiO_2_ nanoparticles were synthesized via the sol–gel method, incorporating Nb^5+^, ions into the TiO_2_ lattice to modulate the structural and electronic properties of TiO_2_ to enhance its photocatalytic performance for CIP degradation under UV and visible irradiation. Comprehensive structural, morphological, and optical analyses revealed that Nb incorporation stabilizes the anatase phase, reduces particle size (from 21.42 nm to 10.29 nm), and induces a slight band gap widening (from 2.85 to 2.87 eV) due to the Burstein–Moss effect. Despite this blue shift, Nb-TiO_2_ exhibited significantly improved photocatalytic activity under visible light, achieving 86% CIP degradation with a reaction rate 16 times higher than that of undoped TiO_2_. This enhancement was attributed to improved charge separation and higher hydroxyl radical (^•^OH) generation, driven by excess conduction band electrons introduced by Nb doping. Density Functional Theory (DFT) calculations further elucidated the electronic structure modifications responsible for this behavior, offering molecular-level insights into Nb dopant-induced property tuning. These findings demonstrate how targeted doping strategies can engineer multifunctional nanomaterials with superior photocatalytic efficiencies, especially under visible light, highlighting the synergy between experimental design and theoretical modeling for environmental applications.

## 1. Introduction

Pharmaceutical pollutants, particularly antibiotics, have emerged as contaminants of global concern due to their persistence in aquatic systems and their potential to induce antimicrobial resistance [1]. Ciprofloxacin (CIP), a widely used fluoroquinolone antibiotic, is frequently detected in rivers, lakes, and drinking water sources at concentrations ranging from nanograms to micrograms per liter [2]. Conventional wastewater treatments have shown inefficiency to altogether remove these low concentrations [2], leading to the accumulation of persistent antibiotic residues that present ecological and human health risks. Photocatalysis has emerged as a promising technology for degrading persistent pollutants, offering an advanced oxidation process (AOP) capable of mineralizing non-biodegradable substances in water, such as pharmaceutical contaminants, including antibiotics, under light irradiation [1,2]. Among the various photocatalysts, titanium dioxide (TiO_2_) is the most widely used photocatalyst due to its high stability [3], non-toxicity [4], and strong oxidative power. TiO_2_ has been commonly used in applications such as gas sensors [5], air and water purification [6,7], dye-sensitized solar cells [8], and self-cleaning photocatalysts [9,10]. However, its wide band gap (~3.2 eV for anatase) limits its activity to the ultraviolet (UV) region, restricting its efficiency under solar and visible light, whilst the rapid recombination of charge carriers (electrons and holes) decreases its photocatalytic efficiency [11]. Various strategies have been developed to overcome these limitations, including doping TiO_2_ with metal and non-metal elements, which has proven effective in tailoring its optical and electronic properties, as well as regulating crystal facet growth to expose more reactive surfaces [12,13,14]. In addition, recent studies highlight that forming heterojunctions, particularly Z-scheme architectures, can significantly enhance the photocatalytic performance [15]. These modifications improve the separation and transfer efficiency of photogenerated charge carriers, enhance the quantum yield, and extend the photoresponse into the visible region, thereby promoting the development of highly efficient visible-light-driven photocatalysts [13,14,15].

For instance, Nd-doped TiO_2_ prepared via sol–gel exhibited a 98% degradation efficiency under visible light [16], whereas Fe-doped TiO_2_ was shown to reduce the band gap, also enhancing the photocatalytic activity [17]. Similarly, nitrogen doping has been reported to extend the absorption edge into the visible range [18]. Niobium (Nb) doping is particularly attractive as it introduces donor levels near the conduction band, thereby altering the Fermi level and enhancing charge separation efficiency [19]. However, despite several experimental studies on Nb-doped TiO_2_, a comprehensive understanding of its impact on charge carrier dynamics, optical transitions, and photo-catalytic mechanisms under visible-light irradiation remains lacking. In this sense, to optimize the material design in terms of the doping agent to improve the photocatalytic activity and to better understand/corroborate the final properties of the synthesized doped TiO_2_ nanoparticles, theoretical studies are required [20]. Liping Wen [21] studied the effect of Fe-doped TiO_2_ in photocatalysis by analyzing the experimental results with DFT. It was found that the experiments and DFT calculation show that the Fe^3+^ ions in bulk TiO_2_ are localized, so they mainly form the recombination centers. Farzad Arjomandi Rad [22] also conducted a combined experimental and DFT study on the structural and photocatalytic properties of nitrogen-doped TiO_2_. It was realized that the nitrogen reduced the band gap of TiO_2_ in both experimental and DFT studies.

Although Nb doping of TiO_2_ has been studied, most research focused on bulk properties and UV-driven activity. By combining experimental and DFT methods, this research, on the other hand, seeks to provide a comprehensive understanding of how Nb doping enhances the photocatalytic efficiency of TiO_2_ nanoparticles. The findings will contribute to developing efficient, visible-light-responsive photocatalysts for water treatment applications, providing a sustainable solution for removing persistent pharmaceutical contaminants such as ciprofloxacin. Additionally, Density Functional Theory (DFT) calculations using CASTEP-2017 softwarewere employed to investigate the electronic structure and absorption properties of Nb-doped TiO_2_, providing deeper insights into band structure modifications and their impact on photocatalytic performance. This computational analysis will complement the experimental findings, enabling a more detailed understanding of the relationship between Nb-induced electronic transitions, charge carrier dynamics, and the photocatalytic behavior of the material.

## 2. Experiments and Computational Methods

### 2.1. Experimental Details

#### 2.1.1. Synthesis of the Nb-Doped Nanocomposites Through Pechini Sol–Gel Process

The precursors for the synthesis are niobium (V) ethoxide (Nb (OCH_2_CH_3_)_5_, Sigma-Aldrich, (99.95%), titanium tetra-isopropoxide (TTIP) solution (C_12_H_28_O_4_.Ti, Sigma-Aldrich), citric acid anhydrous (C_6_H_8_O_7_, Merck), and ethylene glycol (C_2_H_6_O, Merck).

Synthesis details. The Pechini sol–gel process [23] produced Nb-doped TiO_2_. Initially, 6.33 g of citric acid was dissolved in 150 mL of ethanol, and 10 mL of TTIP was also added. The mixture was shaken at room temperature for about an hour to produce a well-dispersed titanium citrate complex solution. Subsequently, the niobium ethoxide levels of 6.25% (molar ratio for Nb and Ti: 6.25:93.75) were combined with the TTIP-citrate solution and stirred at room temperature to form the metal citrate complex. Afterwards, the mixture was stirred for 5 h at 150 °C while adding 30 mL of ethylene glycol. The dried gel was kept in the muffle set at 100 °C for 3 days. Finally, the doped samples were grounded in a mortar and pestle to obtain a fine powder after being calcined for two hours at 500 °C. In addition, pure TiO_2_ was prepared using the same protocol.

#### 2.1.2. Characterizations

The characterization of the produced nanoparticles was performed using various techniques. X-ray diffraction (XRD) patterns of the nanoparticles were obtained with a Panalyticial Cubix (Malvern Panalytical Ltd., Malvern, UK) diffractometer with Cu Kα radiation (λ = 1.5418 Å), with a voltage of 40 kV and a current of 40 mA. The zeta (ζ) potential of TiO_2_ and Nb-TiO_2_ was determined using a Zetasizer Nano ZS (ZEN3600, Malvern Instruments Limited, UK) equipped with an He–Ne laser (633 nm wavelength) and backscatter detection at 173°. Dispersions of 50 mg of nanoparticles (NPs) in 100 mL of ultrapure (UP) water were prepared, and the pH was adjusted to 3, 5, 7, 9, and 11 using 0.5 M HCl or 0.5 M NaOH stock solutions. The measurements followed the Smoluchowski theory [24], with six repetitions for each NP type at 25 °C. The zeta potential values were calculated using Zetasizer 8.02 software. Additionally, the hydrodynamic size of the TiO_2_ nanoparticles was assessed by dynamic light scattering (DLS) using the same Zetasizer NANO ZS-ZEN3600 under the same laser and detection settings. This experiment used the pH 11 solution from the zeta potential measurements, with the nanoparticles tested five times at 25 °C. The hydrodynamic size was also estimated using the Zetasizer 8.02 software.

Additionally, Raman spectroscopy was performed using Renishaw Invia Quontor equipment with a laser excitation wavelength of 532 nm. The X-ray fluorescence spectra (TXRF) were recorded by employing Fischerscope X Ray System SDAL equipment from Fischer brand, operating at 50 KV with an Ni primary filter and a 0.60 Dm collimator. Fourier transform infrared (FTIR) spectroscopy measurements were carried out with a Jasco FT/IR-6100 spectrometer. For FTIR spectra, 64 scans were recorded in the range 4000–400 cm^−1^ with a resolution of 4 cm^−1^. Transmission Electron Microscopy (TEM) images were acquired in a JEOL JEM-1400 PLUS instrument operating at 100 kV, after nanoparticles were deposited on a carbon-coated TEM grid. Field emission scanning electron microscopy (FE-SEM) analyses were carried out using a Hitachi S-4800 at an acceleration voltage of 5 kV. Before imaging, samples were sputtered with a 10-nanometer-thin gold–palladium layer. Images were obtained at magnifications from 50× to 80,000×. The average size of the particles was obtained after measuring more than 150 particles using Image J 2.14.0 software. Absorbance and diffuse reflectance spectroscopy (DRS) were evaluated using a Jasco Analytical UV–Vis–NIR spectrophotometer in the 200 to 2000 nm wavelength range, with measurements taken at 1 nm intervals. No specific correction for light scattering was applied during UV–Vis–NIR measurements, and absorbance data were used directly for band gap estimation. Photoluminescence (PL) spectroscopy was recorded in a Perkin-Elmer LS-55 spectrofluorometer equipped with a xenon lamp at room temperature.

#### 2.1.3. Photochemical Characterization—Quantification of Hydroxyl Radicals (^•^OH)

**Scheme 1:**
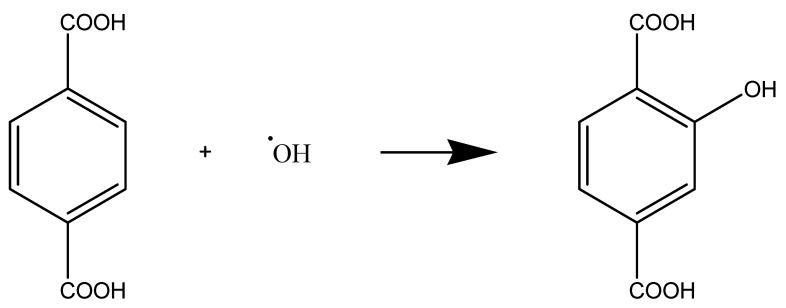
Hydroxylation reaction of terephthalic acid to quantify hydroxyl radicals.

The hydroxylation reaction of terephthalic acid (TA) was employed as a quantification method to determine the concentration of generated ^•^OH under irradiation by following the emission fluorescence spectrum of the hydroxylated product 2-hydroxyterephthalic acid (2-HTA), resulting from the reaction between TA and ^•^OH, according to Scheme 1 [25,26].

In brief, 1.0 g L^−1^ of each synthesized nanomaterial was dispersed into 20 mL of TA solution (5 × 10^−4^ mol L^−1^) and stirred for 2 h in dark conditions. Under UVA irradiation, aliquots of 1 mL were taken at different intervals and filtered through Millipore membranes (0.22 µm). The emission spectra of the supernatant were analyzed using a monochrome tree Infinite M Nano+ at excitation and emission wavelengths of 311 and 425 nm, respectively. Finally, the integrated area of the fluorescence emission picks was related to the ^•^OH concentration through a calibration curve. A control experiment was conducted under identical irradiation conditions without the addition of photocatalysts to validate that hydroxyl radical generation originated only from the photocatalytic process.

#### 2.1.4. Photocatalytic Activity Evaluation

The photocatalytic efficiency of TiO_2_ and Nb-TiO_2_ nanoparticles was tested by photocatalytic degradation of Ciprofloxacin (CIP) under ultraviolet (UV) and xenon (simulated sunlight) radiation. In brief, 50 mg of nanoparticles were added to a beaker containing 50 mL of CIP with a concentration of 10 mg L^−1^, attaining a nanoparticles concentration of 1.0 g L^−1^; this was kept under magnetic stirring for 120 min in dark conditions. The beaker was placed into the photoreactor and vertically irradiated by eight UV lamps (Philips 8 W), with a maximum intensity peak at 365 nm and light intensity of 0.4 mW/cm^2^. Under xenon radiation, the beaker was horizontally irradiated at 15 cm from the lamp, reaching a light intensity of 27 mW/cm^2^. The photocatalytic efficiencies were analyzed by monitoring the maximum absorption peak (273 nm) of CIP over time of irradiation, using an Infinite M-Nano+ UV/Vis spectrophotometer.

### 2.2. Computational Methods

Using the ultra-soft pseudo potential approach implemented in the CASTEP code and the Perdew–Burke–Ernzerhof (PBE) exchange-correlation potential, the calculations were carried out within the Density Function Theory (DFT) framework in the generalized gradient approximation (GGA) [27]. The computations of the pseudo atomic states were carried out for Ti (3d^2^4s^2^), O (2s^2^2p^4^), and Nb (4d^4^5s1). The cut-off for the plane-wave basis set was 520 eV. A TiO_2_ and Nb-doped 2 × 2 × 1 Monkhorst-Pack k-point grid was used to optimize the geometry [28]. One niobium atom was substituted for one Ti atom to create the Nb-doped configurations. The atomic and cell relaxations were performed until the atomic displacement was less than 5 × 10^−4^ Å, the convergence threshold of total energy was less than 5 × 10^−6^ eV/atom, the residual forces were less than 0.01 eV/Å, and the stress on the atoms was less than 0.02 GPa. The electronic and optical properties and elastic constants were computed.

## 3. Results and Discussion

### 3.1. Physical-Chemical Properties

**Figure 1:**
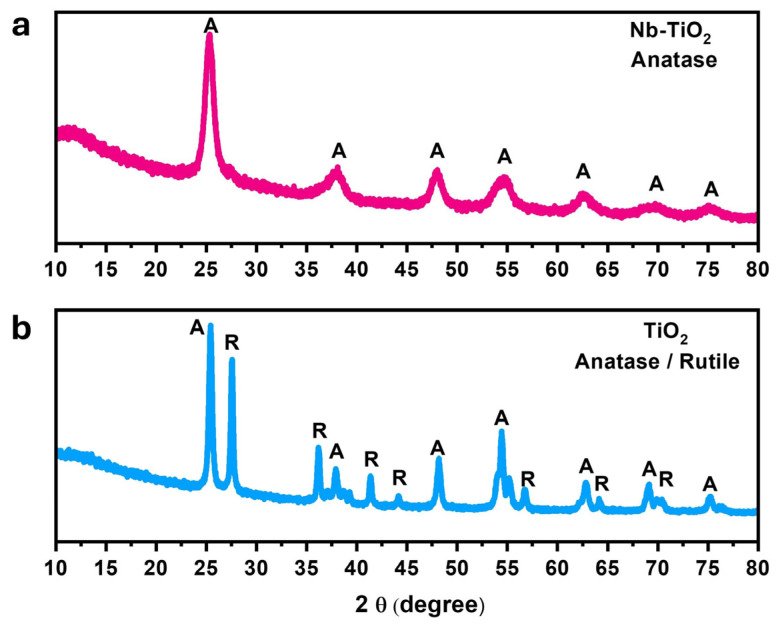
X-ray diffraction patterns of (**a**) Nb-doped and pure (**b**) TiO_2_ nanoparticles (A: Anatase, R: Rutile).

XRD was used to determine the phase composition of both Nb-doped TiO_2_ and the pure TiO_2_ nanoparticles. As shown in Figure 1a, the Nb-doped sample exhibits diffraction peaks exclusively associated with the anatase phase. The peaks correspond to the (101), (004), (200), (105), (204), (116), and (215) crystallographic planes at 25.28°, 38.03°, 47.93°, 54.54°, 62.62°, 69.57°, and 75.16°, respectively, in agreement with the pattern (JCPDS 84-1286) [29]. In contrast, Figure 1b reveals that the undoped TiO_2_ sample presents a mixed phase of anatase and rutile. The rutile phase is confirmed by the presence of peaks at 2θ = 27.53°, 36.21°, 41.19°, 44.21°, 56.72°, 64.14°, and 70.28°, corresponding to the (110), (101), (111), (210), (220), (310), and (301) planes, respectively, in agreement with JCPDS card No. 75-1753 [30]. The anatase-to-rutile ratio was estimated using equations based on the relative intensities of the anatase (101) and rutile (110) peaks using Equations (1) and (2) [31]:

(1) $$WA=\;\frac{{0.886I}_{A}}{{0.886I}_{A}+I_{R}}$$

(2) $$WR=\frac{{0.886I}_{R}}{{0.886I}_{R}+I_{A}}$$

where W_A_ and W_R_ denote anatase and rutile TiO_2_ weight percentages, respectively, I_A_ and I_R_ represent the respective peak intensities. The obtained phase content was W_A_ = 52% and W_R_ = 42%, indicating anatase as a major phase present in the TiO_2_ sample. This distinct phase difference between the doped and undoped samples is consistent with previous findings and can be attributed to the stabilizing effect of Nb^5+^ of the anatase structure [32,33,34]. Nb^5+^ substitutionally incorporated into the Ti^4+^ sites introduces local lattice strain, stabilizes defect structures, and alters the charge compensation mechanisms, thereby raising the energy barrier required for the structural rearrangement of the titanium and oxygen ions necessary for the anatase-to-rutile phase transformation [32,33,34]. According to Arbiol et al. [33], Nb^5+^ incorporation introduces localized strain and reduces the oxygen vacancy concentration, being two key factors that kinetically hinder the transformation of anatase to rutile during thermal treatment. In this study, high-resolution TEM and Raman analysis showed that even small Nb loadings (~2.9 at.%) significantly delay the onset of rutile formation up to temperatures > 850 °C. Further supporting evidence from Mardare and Hones [32] shows that Nb-doped TiO_2_ thin films (0.35 at.%) crystallize purely in the anatase phase under low-temperature deposition conditions (250 °C), whereas undoped films tend to form a mixed anatase/rutile structure. This highlights the structural role of Nb^5+^ in modifying the phase evolution path of TiO_2_, even at relatively low concentrations. Additionally, Zhang and Banfield [34] emphasized that the anatase-to-rutile phase transformation is both size and defect controlled. They demonstrated that smaller particles (<14 nm) thermodynamically favor the anatase phase due to surface energy effects, while grain growth is a prerequisite for rutile nucleation. In our study, Nb-doped TiO_2_ exhibited a smaller crystallite size, as determined by the Scherrer equation (Equation (3)), which further contributes to the stabilization of anatase by suppressing the grain growth required for the rutile phase transformation.

The crystallite size (D) was determined by applying the Scherer equation to the XRD diffraction data [35]:

(3) $$D=\frac{K\lambda \;}{\beta \cos\theta }$$

where D represents the mean size of the nano crystallite (nm), k = 0.94 (Scherer constant), λ denotes the wavelength of the X-ray radiation (Cu Kα = 0.15406 nm), β represents the full width at half-maximum intensity of the diffraction peak measured at 2θ (in radians), and θ stands for the diffraction angle. For the doped and the pure samples, the average crystalline sizes are shown in Table 1, showing that the crystallite size decreases with the Nb doping. This size effect, in combination with the electronic and defect-structural modifications induced by Nb incorporation, provides a comprehensive explanation for the absence of rutile in the doped sample despite identical calcination conditions (500 °C, 2 h) [17].

To further confirm Nb^5+^ incorporation into the TiO_2_ lattice, lattice parameters were calculated using Bragg’s law for a tetragonal system (Equation (4)), using the (101) and (200) diffraction planes [36], where h, k, and l are Miller’s indices for the crystallographic plane. As reported in Table 1, both the a and c lattice parameters and unit cell volume are slightly increased in the doped sample. This shift is attributed to the larger ionic radius of Nb^5+^ (0.64 Å) compared to Ti^4+^ (0.605 Å) [37] and aligns with prior findings [38], demonstrating the successful substitutional doping of Nb^5+^ into the crystal lattice of TiO_2_, as previously demonstrated for other dopants such as neodymium Nd^+3^ ions [39].

(4) $$\frac{1}{d_{hkl}^{2}}=\left(\frac{h^{2}+k^{2}}{a^{2}}\right)+\frac{l^{2}}{c^{2}}$$

**Figure 2:**
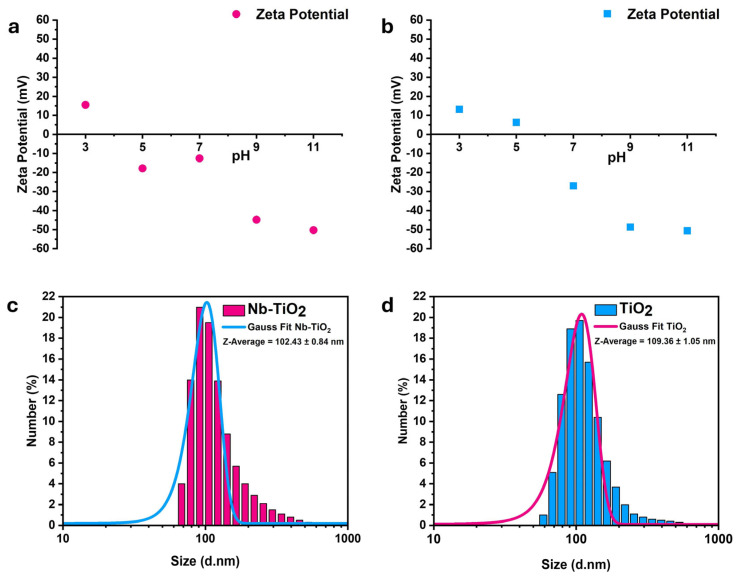
Zeta potential graphs of (**a**) Nb-TiO_2_ and (**b**) pure TiO_2_. (**c**) Hydrodynamic size distributions of (**c**) Nb-TiO_2_ and (**d**) pure TiO_2_ nanoparticles.

Zeta potential measurements allowed estimating the periphery surface charge of the Nb-TiO_2_ and TiO_2_ nanoparticles in the pH 3 to pH 11 range—Figure 2a,b. Both particles show similar profiles, with an estimation of isoelectric points between pH = 3.5 and pH = 4 for Nb-TiO_2_ and between pH = 5 and pH = 5.4 for TiO_2_ nanoparticles, showing that the presence of Nb causes a decrease in the isoelectric point, which is consistent with the low pHs for isoelectric points estimated for Nb in other studies [40,41].

Additionally, in both samples, the highest zeta potential values, around −50 mV, are obtained at pHs between 9 and 11. This indicates that the nanoparticles are more stable in solution under these basic pHs, as the higher peripheric charges promote repulsions between nanoparticles. Further, the average hydrodynamic size of the nanoparticles was ascertained by dynamic light scattering (DLS) on both samples at pH = 11, as shown in Figure 2c,d.

The DLS results show that the average sizes of the Nb-TiO_2_ and TiO_2_ nanoparticles are 102 nm and 109 nm, respectively, suggesting that the addition of Nb has no discernible effect on the behavior of TiO_2_ in solution. The polydispersity indexes (PDIs) for TiO_2_ and Nb-TiO_2_ are 0.6 and 0.5, respectively. The comparatively large PDIs raise the possibility of an aggregation phenomenon or a wide size dispersion of the nanoparticles.

**Figure 3:**
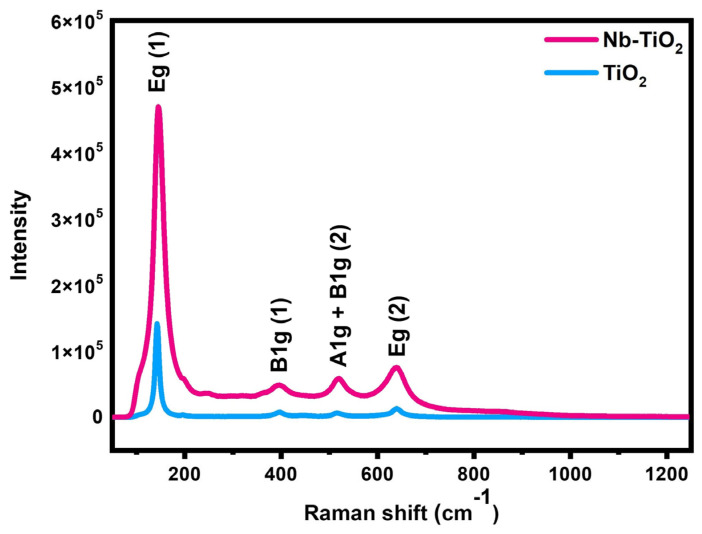
Raman spectroscopy of Nb-doped and pure TiO_2_ nanoparticles.

**Figure 4:**
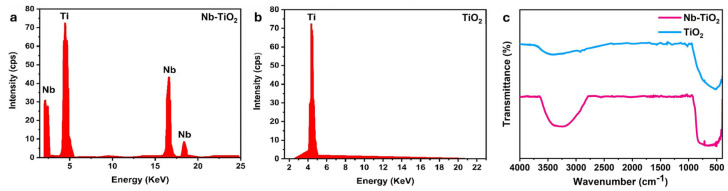
(**a**,**b**) X-ray fluorescence and (**c**) FTIR spectra of Nb-doped and undoped TiO_2_ nanoparticles.

Surface structural variations were obtained by Raman analysis both before and after doping between 0 and 1200 cm^−1^, as represented in Figure 3. The Raman active modes Eg (1), B1g (1), A1g + B1g (2), and Eg (2) are represented by the detected bands at 141.51, 397.26, 517.36, and 641.97 cm^−1^, respectively. All these modes are for the anatase phase of TiO_2_ [42]. The Eg mode’s Raman peak positions are 146.03 cm^−1^ for Nb-TiO_2_ and 144.51 cm^−1^ for TiO_2_. This variation can be attributed to the observed variations in crystal size [43]. X-ray fluorescence elemental analysis was carried out to confirm the incorporation of Nb in the TiO_2_ lattice. As shown in Figure 4a,b, the undoped TiO_2_ sample exhibits a high Ti content of 99.86 wt.%, with 0.14% impurities, while the Nb-doped TiO_2_ presents a measurable Nb content of 2.66 wt.%, in close agreement with the theoretical loading used during synthesis. This confirms the incorporation of Nb into the structure. While XRF cannot directly detect oxygen atoms [44], it provides reliable quantitative elemental data to validate substitution in the lattice.

The FTIR spectra of TiO_2_ and Nb-TiO_2_ displayed in Figure 4c show a characteristic absorption band near 3422 cm^−1^ corresponding to O-H stretching of surface hydroxyl groups [45,46]. In this region, the Nb-doped sample exhibits more intense and broader O-H bands, indicating an increased concentration of Brønsted acid sites upon Nb incorporation [46]. This enhancement suggests that Nb doping promotes surface hydroxylation, consistent with previous findings linking Nb substitution to the transformation of acidic sites [45]. Additionally, the broad absorption in the 450–700 cm^−1^ region that appears in both samples is mainly associated with Ti-O bending vibrations, [45]. Although the Nb-TiO_2_ spectrum does not exhibit a distinct Nb-O peak due to spectral overlap with Ti-O modes, the intensity of the Ti-O-Ti vibrations increases around 600 cm^−1^, and broad O-H vibrations support the hypothesis of increased acidic character, as reported previously [45].

**Figure 5:**
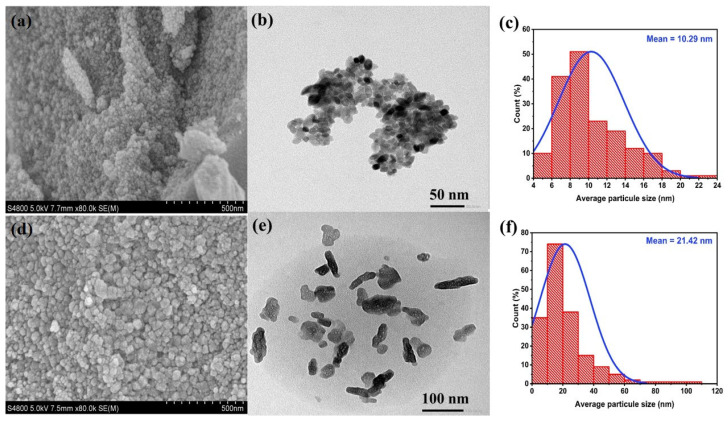
SEM images of (**a**) Nb-TiO_2_ and (**d**) pure TiO_2_ nanoparticles. (**b**,**e**) TEM images for the respective samples. (**c**,**f**) Histograms of the crystalline sizes for Nb-TiO_2_ and pure TiO_2_.

The SEM and TEM analyses of the synthesized Nb-doped TiO_2_ and undoped TiO_2_ nanoparticles are shown in Figure 5a,b,d,e. The analyzed images reveal clear morphological differences between both samples. Nb-doped TiO_2_ in Figure 5a,b consists of smaller, more homogeneously distributed nanoparticles, predominantly spherical in shape, with sizes ranging from 4 to 20 nm, as supported by the size distribution histogram in Figure 5c, which indicates an average particle size of 10.29 nm. These doped particles exhibit a relatively isotropic shape and low aspect ratio, indicating a more controlled nucleation and suppressed anisotropic growth, likely due to the structural distortions induced by Nb^5+^ incorporation into the TiO_2_ lattice. In contrast, undoped TiO_2_ nanoparticles in Figure 5d,e are larger particles, less uniformly distributed with a tendency to aggregate, forming dense clusters. The TEM image in Figure 5e reveals elongated particles with sizes between 5 and 60 nm, whereas the particle size distribution histogram in Figure 5f shows a significantly larger average size of 21.42 nm. This elongated morphology suggests faster growth along specific crystallographic planes. The comparative analysis suggests that Nb incorporation affects not only the crystallization dynamics but also the surface energy and growth rates of specific crystal facets, preventing excessive aggregation that results in finer and more uniform nanoparticles.

The observed decrease in particle size upon Nb incorporation aligns with the Scherrer estimation of average crystalline size. This reduction in particle size is attributed to the substitution of Ti^4+^ by Nb^5+^ within the TiO_2_ lattice. Since Nb^5+^ has a slightly larger ionic radius (0.64 Å) compared to Ti^4+^ (0.605 Å), its incorporation induces lattice distortions and creates structural defects that act as barriers to grain growth. These distortions create energy barriers that hinder grain boundary mobility, thereby limiting crystal growth and resulting in smaller nanoparticles [47]. This phenomenon has been previously reported in studies on Nb-doped TiO_2_, where increasing Nb doping levels have been correlated with a progressive decrease in grain size due to defect-induced growth suppression [33,47]. Thus, Nb doping significantly modifies the particle morphology and size of TiO_2_, leading to the formation of smaller and more homogeneously distributed nanoparticles, demonstrating its influences on the microstructural characteristics of the nanomaterial.

**Figure 6:**
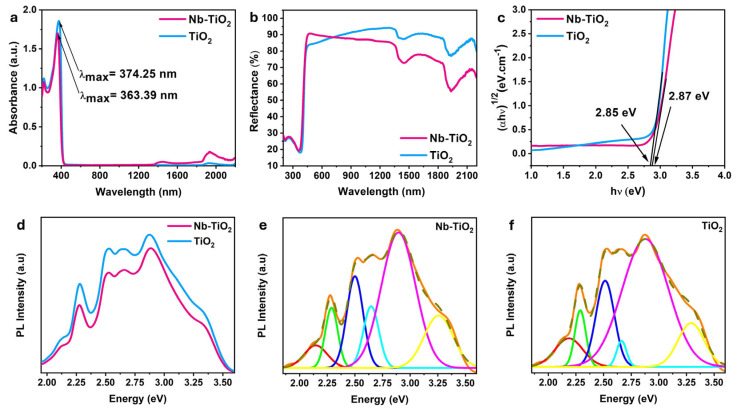
(**a**,**b**) Absorbance and reflectance spectra, (**c**) (hν)^1/2^ versus hν plots, (**d**) PL emission spectra under 254 nm, and (**e**,**f**) deconvoluted PL emission spectra for Nb-doped and pure TiO_2_ nanoparticles.

The absorbance and diffuse reflectance spectra (DRS) of the synthesized particles recorded between 200 and 2200 nm are represented in Figure 6a,b. As observed in the absorbance spectra, both samples exhibit strong absorption in the UV region, corresponding to the intrinsic transition of the electrons from the valence band to the conduction band of these semiconductors [48]. The maximum absorbance peak shifts slightly from 363.39 nm for TiO_2_ to 374.25 nm for Nb-TiO_2_, showing that Nb incorporation affects the optical properties of the material. This redshift in the absorption edge indicates modifications in the electronic structure due to the introduction of Nb^5+^ ions. The DRS were used to determine the energy band gap of the materials. Thus, the optical band gap of the nanoparticles was obtained from the Tauc plot against incident light using Equation (5) [43,49,50]:

(5) (αhv)^1/2^ = A (hv − Eg)

where hv represents the photon energy, Eg is the energy band gap, A is a constant, and the absorption coefficient is represented by α (α = 4πk/λ, where λ is the wavelength in nm and k is the absorbance index).

The indirect Eg was determined by extrapolating the linear region of (αhv)^1/2^ = 0 vs. hv, as represented in Figure 6c. The band gaps for the Nb-doped and TiO_2_ samples are 2.87 eV and 2.85 eV, respectively. The observed reduction in the band gap to 2.85 eV, compared to the typical 3.2 eV for anatase TiO_2_, suggests that additional mechanisms are at play, in particular, defects and lattice distortion in the crystalline structure [51]. In the case of the doped material, the narrow band gap is attributed to a combination of Nb doping, and potential lattice distortions [51,52]. These modifications introduce new electronic states within the band structure, facilitating enhanced absorption of visible light and improved photocatalytic performance. Moreover, the larger ionic radius of Nb^5+^ (0.64 nm) compared to Ti^4+^ (0.605 nm) can lead to lattice strain, further altering charge transport and optical absorption [53]. Despite the possibility that Nb doping could lead to an Eg narrowing, a slight increase in the band gap was observed for the doped sample. The incorporation of Nb^5+^ ions into the TiO_2_ lattice affects not only its crystalline phase and particle size but also its electronic structure by introducing donor levels near the conduction band [19], resulting in a shift of the Fermi level closer to the conduction band [52]. This shift can increase the semiconductor band gap due to the Burstein–Moss effect [54], where the elevated electron concentration fills the lower energy states in the conduction band, pushing the absorption edge to higher energies. Thus, the slight observed blue shift is mainly attributed to Nb^5+^ doping-induced electronic structure modifications, rather than an increase in oxygen vacancies. This effect is further supported by the lower intensity of oxygen-vacancy-related PL emission in the Nb-doped sample discussed below, suggesting a reduced presence of mid-gap states and confirming that the band gap widening is mainly due to donor-induced Fermi level shifts rather than a narrowing driven by oxygen vacancies (OVs) [55].

On the other hand, PL analysis was performed to investigate the nature of structural defects and their influence on the electronic band structure upon Nb doping. In TiO_2_-based materials, PL emission is typically associated with self-trapped excitons, oxygen vacancies (OVs), and surface defect states, which can introduce localized energy levels within the bandgap and thus modify their optoelectronic behavior [55]. The PL spectra recorded under 254 nm excitation (Figure 6d) exhibit a broad emission band in the 350–650 nm range, extending well into the visible region, with a clear observed difference in the emission intensity between pure TiO_2_ and Nb-doped TiO_2_. To gain further insight, both spectra were deconvoluted using Gaussian fitting into six distinct emission peaks (Figure 6e,f). The broad violet and blue emission bands centered at 3.35 eV (371 nm) and 2.90 eV (428 nm) are attributed to self-trapped excitons at TiO_6_ octahedra and are indicative of electron–hole recombination processes. Herein, the reduced PL intensity observed in the Nb-doped TiO_2_ sample compared to undoped TiO_2_ suggests a lower recombination rate of photogenerated charge carriers [48]. This observation implies enhanced charge separation and supports the role of Nb^5+^ as an electron donor, rather than merely promoting OV formation [55], contributing to increased electron density and potentially facilitating higher ROS generation for improving photocatalytic activity [48]. Additionally, the green emission peaks located at 2.67 eV (465 nm), 2.53 eV (491 nm), and 2.29 eV (542 nm) are attributed to oxygen vacancy defect-related states located just below the conduction band [55,56]. A clear decrease in the intensity of these defect-related PL bands is observed for the Nb-doped TiO_2_ sample compared to the undoped one (Figure 6e,f), implying a lower concentration of OVs in the doped material. This reduction is attributed to the substitution of Ti^4+^ by Nb^5+^ ions, which presents a higher positive charge. To keep the local charge balance in the lattice, the formation of oxygen vacancies is suppressed [33], resulting in decreased OV-related emission signals.

### 3.2. Photocatalytic Efficiency of Nb-TiO_2_ and TiO_2_ Nanoparticles

**Figure 7:**
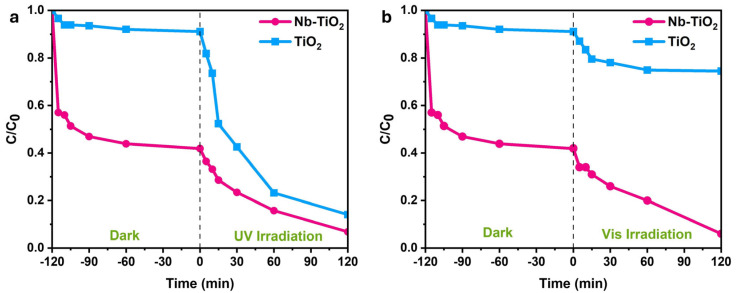
Removal of CIP (10 mg/L) after 240 min of contact with Nb-TiO_2_ and pure TiO_2_ (**a**) under UV irradiation and (**b**) under visible irradiation for 120 min.

The photocatalytic degradation of ciprofloxacin (CIP) using Nb-TiO_2_ and TiO_2_ nanoparticles was investigated under UV and visible irradiation, as shown in Figure 7. Before irradiation, the adsorption equilibrium was established during 120 min in the dark. The results indicate a significant difference in the adsorption behavior of Nb-TiO_2_ compared to undoped TiO_2_. Nb-TiO_2_ exhibited a high adsorption capacity, removing ~61% of CIP before irradiation, whereas TiO_2_ adsorbed only ~9%, as shown in Figure 7a,b. This indicates that Nb incorporation enhances surface interaction with CIP molecules, likely due to Nb providing numerous Brønsted acid sites to the TiO_2_-based material [57], thereby modifying its surface chemistry, as demonstrated by zeta potential and FTIR measurements, which promote higher interaction between the material’s surface and CIP molecules [58].

Afterwards, under UV irradiation, Figure 7a shows that TiO_2_ exhibits a slightly higher photocatalytic degradation rate than Nb-TiO_2_ after 120 min of irradiation, with 84% of degradation compared to 83% degradation, respectively. Nevertheless, after accounting for both processes (adsorption and photo-oxidation), the total removal of CIP by Nb-TiO_2_ reached 93%, while TiO_2_ reached 86%. On the other hand, under visible light (Figure 7b), a strong difference in the photocatalytic performance is observed. Under these conditions, Nb-TiO_2_ photo-oxidized 86% of CIP after 120 min, while TiO_2_ achieved only 18% degradation. Thus, after both processes, adsorption under dark and oxidation under visible light, the total removal efficiency reached for Nb-TiO_2_ was 94% and 26% for TiO_2_. This suggests that Nb doping enhances visible-light absorption and charge separation efficiency, boosting photocatalytic activity under visible irradiation. Although the present study employed only UV–VIS spectroscopy to monitor CIP degradation, it is important to note that in previous studies, liquid chromatography–mass spectrometry (LC–MS) analyses have been used to assess mineralization pathways and degradation intermediates in detail [50,59].

**Figure 8:**
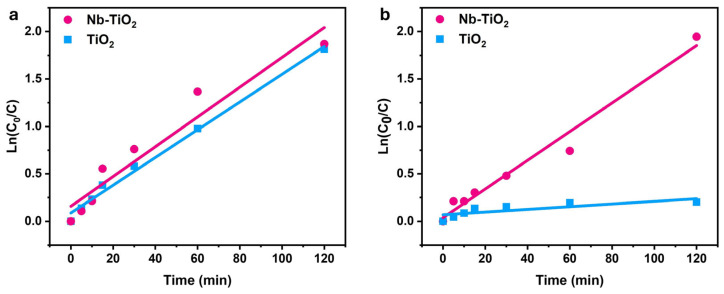
Pseudo-first kinetic model for CIP degradation by photocatalyst for Nb-TiO_2_ and TiO_2_ (**a**) under UV irradiation and (**b**) under visible irradiation.

The kinetic curves of CIP photodegradation were fitted using the pseudo-first-order kinetics mode given by Equation (6):

(6) $$\ln(C_{0}/C_{t})=k_{app}.t$$

where C_0_ and C_t_ are the initial and final concentrations of CIP solution at irradiation time t, respectively, and k_app_ corresponds to the apparent rate constant. The linearity of the plots in Figure 8 demonstrates that the degradation follows a pseudo-first-order reaction mechanism, and Table 2 presents the k_app_ values and the corresponding linear regression coefficients. Significant differences in the rates of CIP degradation between both photocatalysts were observed, demonstrating the effect of Nb incorporation on the improved photoactivity under visible light. Under UV irradiation, the k_app_ values are 0.014 min^−1^ and 0.016 min^−1^ for the Nb-TiO_2_ and TiO_2_ nanoparticles, respectively. The slight decrease in k_app_ for Nb-TiO_2_ can be attributed to Nb-induced structural distortions that limit UV absorption efficiency [60]. In contrast, despite the increased band gap in the Nb-TiO_2_ sample, under visible irradiation, the doped sample shows a significantly higher rate constant (0.015 min^−1^), demonstrating its superior visible-light photo-activity. TiO_2_ nanoparticles exhibits lower k_app_ value (0.001 min^−1^), confirming their poor response to visible radiation. These findings confirm that Nb doping significantly enhances the visible-light photocatalytic activity of TiO_2_ due to band structure modifications and improved charge carrier separation, since Nb can trap the photogenerated electron–hole pairs, preventing their recombination, which increases the overall photocatalytic activity [61]. In addition, the presence of a 100% anatase crystalline phase in the Nb-doped TiO_2_ sample could also boost this observed enhancement, as anatase is known to be the most active phase in photocatalytic reactions.

#### Photochemical Mechanism: Hydroxyl Radical Generation

**Figure 9:**
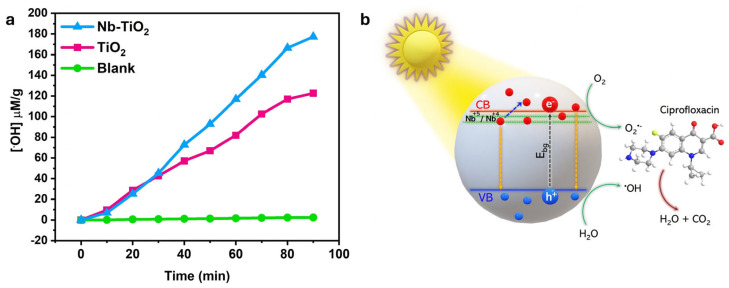
(**a**) Quantification of hydroxyl radicals generated by photocatalysis of Nb-doped and TiO_2_ samples, (**b**) photocatalytic mechanism of CIP degradation on Nb-doped TiO_2_.

The observed enhancement in the photocatalytic performance of Nb-doped TiO_2_ is closely linked to its photochemical behavior, including the generation of reactive oxygen species (ROS), such as ^•^OH, superoxide radical anion (O_2_^−•^), and singlet oxygen (^1^O_2_), among others, and the underlying mechanisms that govern charge carrier dynamics. Upon irradiation with photons having energy equal to or greater than the bandgap of the material, photogenerated electrons (e^−^) are promoted from the valence band (VB) to the conduction band (CB), leaving behind positively charged holes (h^+^) in the VB. These charge carriers drive redox reactions at the surface [62], producing ROS including ^•^OH radicals, as shown in Figure 9a, which are the main oxidizing species in photocatalytic processes according to the overall photochemical process presented in the schematic mechanism of Figure 9b [26,53].

As shown in Figure 9a, Nb-doped TiO_2_ produces significantly higher concentrations of ^•^OH radicals under UVA irradiation compared to undoped TiO_2_. The superior generation of ^•^OH, attributed to synergistic effects induced by Nb^5+^ doping, confirms the efficiency of the Nb-TiO_2_ photocatalyst in initiating ROS-mediated degradation reactions, enhancing both photoinduced charge separation and surface redox activity, and consequently boosting photocatalytic efficiency under both UV and visible irradiation. Herein, Nb^5+^ substitutionally replaces Ti^4+^ in the anatase TiO_2_ lattice, introducing extra electrons and donor energy levels near the conduction band, increasing free electron density and electrical conductivity of the material. These donor states promote visible-light absorption by enabling sub-bandgap excitation and act as shallow traps that extend charge carrier lifetimes, reducing electron–hole recombination [63], as was observed in the PL analysis. In addition, the enhanced electron mobility given by the incorporation of Nb^5+^ creates an internal electric field that drives spatial separation of photogenerated charge carriers, facilitating their transport to the solid surface. Moreover, Nb doping modifies the surface acidity and increases the number of Brønsted acid sites, promoting the interaction and stronger adsorption of polar molecules like ciprofloxacin [63]. Thus, the combination of enhanced charge carrier separation, increased electron mobility, and optimized surface acidity led to the generation of higher concentrations of ^•^OH radicals, resulting in superior photocatalytic performance.

## 4. Theoretical Results

### 4.1. Electronic Properties

**Figure 10:**
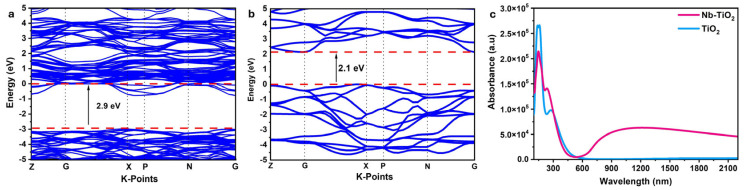
(**a**,**b**) Band structures by CASTEP software for Nb-TiO_2_ and pure TiO_2_, respectively; (**c**) theoretical absorbance spectra of Nb-TiO_2_ and pure TiO_2_.

To fully comprehend the band structure and investigate the impact of Nb’s electronic structures on the surface of TiO_2_, the GGA-PBE functional was utilized to compute the band gap of Nb-TiO_2_, as shown in Figure 10a,b.

Figure 10a shows that the predicted band gap of Nb-doped TiO_2_ is 2.9 eV, in agreement with the experimental results presented in Figure 6c. Additionally, theoretical calculations confirm that niobium doping increases the band gap of pure TiO_2_. The computed band gap for undoped TiO_2_ is 2.1 eV, as shown in Figure 10b, which is consistent with previous theoretical studies [64]. In contrast, the experimentally determined band gap for anatase TiO_2_ is approximately 3.2 eV [65], with the well-known underestimation of density functional theory (DFT) calculations accounting for this discrepancy.

A key finding, as illustrated in Figure 10a, is that the band gap increases upon Nb doping, reaching 2.9 eV. This trend aligns with both theoretical and experimental observations, as shown in Figure 6c, and is supported by prior computational results [66]. The widening of the band gap occurs as Nb atoms substitute Ti atoms, leading to the formation of impurity states primarily composed of Nb 4d orbitals. Consequently, the valence and conduction bands shift downward and broaden. This effect is characteristic of transition metal doping in metal oxides, where the introduction of dopant states influences electronic structure and optical properties.

### 4.2. Optical Properties

Figure 10c shows the theoretical absorbance spectrum of Nb-doped and TiO_2_ samples. Note that both experimental Figure 6a and theoretical figures demonstrate that the absorption spectra of both samples are mainly located in the ultraviolet spectrum. The theoretical and experimental results of TiO_2_ in reference [67,68] are consistent with this. It can be also noted that after Nb doping of TiO_2_, the sample had better absorbance under visible radiation than TiO_2_. Moreover, this could explain the enhancement of photocatalytic activity in this region.

## 5. Conclusions

This study presented a novel combined experimental and theoretical approach showing how Nb doping enhances the photocatalytic activity of TiO_2_. In this research, both Nb-TiO_2_ and pristine TiO_2_ were synthetized via the sol–gel method, with the doped sample exhibiting anatase phase stabilization, reduced particle size, and improved surface charge properties. Structural analysis indicated a notable decrease in particle size from 21.42 nm for undoped TiO_2_ to 10.29 nm for Nb-TiO_2_ due to the incorporation of Nb^5+^, leading to lattice distortions that impeded crystal growth. Furthermore, by improving charge separation, the CIP degradation of Nb-TiO_2_ under visible light was 86%, which is a significant improvement over the 18% for undoped TiO_2_. Indeed, both materials exhibited comparable performance under UV irradiation, with nearly total CIP removal. Ultimately, DFT calculations confirmed these results, demonstrating that Nb^5+^ creates donor states near the conduction band, which promotes charge separation and marginally widens the band gap. Simulated absorbance spectra closely matched experimental data, reinforcing the observed optical modifications. Overall, the combined experimental and theoretical results emphasize Nb-TiO_2_ as a suitable material for sustainable water purification applications, providing a practical approach for the removal of pharmaceutical pollutants.

## Figures and Tables

**Table 1:** Lattice parameters and the average crystallite sizes of TiO_2_ and Nb-TiO_2_.

Samples	a = b (Å)	C (Å)	Average D (nm)	Volume V (Å^3^)
Nb-TiO_2_	3.78	9.5	4.69	135.73
TiO_2_	3.77	9.47	13.64	134.59

**Table 2:** Apparent pseudo-first-order reaction rate constant and the linear regression coefficients (R^2^) for CIP photodegradation by the synthesized TiO_2_-based nanomaterials.

Samples	Under UV Irradiation		Under Vis Irradiation	
	*k_app_* × 10^−2^ (min^−1^)	R^2^	*k_app_* × 10^−2^ (min^−1^)	R^2^
Nb-TiO_2_	1.4	0.99	1.5	0.98
TiO_2_	1.6	0.96	0.1	0.79

## Data Availability

Data will be made available on request.
